# Modeling Human Cardiac Thin Filament Structures

**DOI:** 10.3389/fphys.2022.932333

**Published:** 2022-06-22

**Authors:** Michael J. Rynkiewicz, Elumalai Pavadai, William Lehman

**Affiliations:** Department of Physiology and Biophysics, Boston University School of Medicine, Boston, MA, United States

**Keywords:** tropomyosin, actin, troponin, cryoEM, protein-protein docking

## Abstract

Striated muscle contraction is regulated in a calcium-dependent manner through dynamic motions of the tropomyosin/troponin polymer, a multicomponent complex wrapped around actin-containing thin filaments. Tropomyosin/troponin sterically blocks myosin-binding at low-calcium concentrations but moves to expose myosin-binding sites at high-calcium concentrations leading to force development. Understanding the key intermolecular interactions that define these dynamic motions will promote our understanding of mutation-induced contractile dysfunction that eventually leads to hypertrophic cardiomyopathy, dilated cardiomyopathy, and skeletal myopathies. Advancements in cryoelectron microscopy (cryoEM) have resulted in a partial elucidation of structures of the thin filament, revealing many atomic-level interactions between the component proteins and critical calcium-dependent conformational alterations. However, building models at the resolutions achieved can be challenging since landmarks in the maps are often missing or ambiguous. Therefore, current computational analyses including *de novo* structure prediction, protein-protein docking, molecular dynamics flexible fitting, and molecular dynamics simulations are needed to ensure good quality models. We review here our efforts to model the troponin T domain spanning the head-to-tail overlap domain of tropomyosin, improving previous models. Next, we refined the published cryoEM modeled structures, which had mistakenly compressed alpha helices, with a model that has expected helical parameters while matching densities in the cryoEM volume. Lastly, we used this model to reinterpret the interactions between tropomyosin and troponin I showing key features that hold the tropomyosin cable in its low-calcium, sterically blocking position. These revised thin filament models show improved intermolecular interactions in the key low- and high-calcium regulatory states, providing novel insights into function.

## Introduction

**FIGURE 1:**
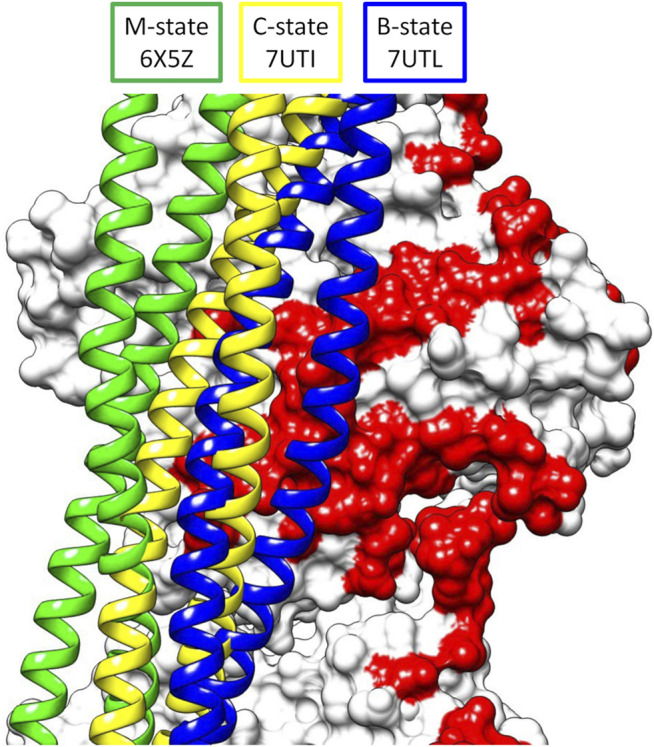
Superposition of the three structural states of tropomyosin during thin filament activation of the M-state (green), C-state (yellow), and B-state (blue) showing the movement of tropomyosin over actin (white surface, pointed end up). Note the steric blocking of the myosin binding site (red) is relieved as tropomyosin moves from B- to M-state.

The improvements in methodology of cryoelectron microscopy (cryoEM) have pushed the limit of resolution achievable by this technique to unprecedented levels, allowing visualization of previously unseen structural details in the thin filament of the sarcomere in striated muscle (Doran et al., 2020; Yamada et al., 2020; Risi et al., 2021a; Risi et al., 2021b; Wang et al., 2021; Wang et al., 2022). These thin filaments consist of actin, tropomyosin, and the troponin complex whose structural transitions regulate contraction in a calcium-dependent manner (reviewed in Lehman, 2016). Tropomyosin is an alpha-helical, coiled-coil protein that forms a head-to-tail polymeric cable wrapped helically around the central actin core of the filament (Hitchcock-DeGregori, 2008; Holmes and Lehman, 2008). The head-to-tail region stabilizing the polymer is formed by an overlap between adjacent tropomyosin dimers of about 11 amino acids where the coiled coil becomes a 4-helix bundle (Greenfield et al., 2006; Frye et al., 2010). The troponin complex consists of three proteins—troponin C (TnC), troponin I (TnI), and troponin T (TnT) (Gordon et al., 2000). TnC along with the N-terminal domains of TnI and the C-terminal domains of TnT form a central core (Takeda et al., 2003; Yamada et al., 2020; also reviewed in Tobacman, 2021). The N-terminal domains of TnT extend from the core to anchor the troponin complex to tropomyosin. Under low calcium conditions, the C-terminus of TnI extends outward from the core where it binds actin and tropomyosin, thus locking the cable in a blocked state position that sterically inhibits myosin access to the thin filament leading to relaxation of the muscle. Higher calcium concentrations lead to conformational changes in calcium-bound TnC that recruit TnI back to the core, releasing the steric block and forming a closed state. Myosin binding to the closed state then results in a further shift of the cable to an open state leading to contraction of the muscle. Thus, the thin filament accesses three structural states—B-state (blocked), C-state (closed), and M-state (open) in its regulatory transitions, as originally proposed by McKillop and Geeves (1993) and by Vibert et al. (1997). All three of these states have now been elucidated by cryoEM (Doran et al., 2020; Yamada et al., 2020) using cardiac muscle filaments (Figure 1).

The development of inherited cardiomyopathies starts with an insult to the contractile machinery that alters the force characteristics of the heart and eventually leads to pathological remodeling (Deranek et al., 2019). Therefore, these cryo-EM structures offer vast potential to increase our understanding of the genesis of cardiomyopathies or to develop novel therapeutics. However, it is critical that accurate atomic-level details are incorporated into the models of the thin filament being used. Otherwise, errors in the structure will propagate into the predictions, leading to misleading or incorrect data, and impeding efforts to develop therapeutics. As will be shown, some regions of the thin filament structure require extra consideration to achieve this needed accuracy.

## Modeling Cardiac Thin Filament Structures From Cryoelectron Microscopy Electron Density Maps

### Challenges of Interpreting Electron Density Maps

A quality structure should not only fit the cryoEM density map well but have good chemical parameters, including optimized interactions between proteins or domains, and ideally representing the global energy minimum structure. Unfortunately, the resolutions achieved in the current cryoEM structures may be insufficient to unambiguously build a model in some sections of the electron density map. While programs such as MolProbity (Chen et al., 2010) can be used to check the bonds, angles, and backbone/side chain dihedrals of a structure, they do not check the reasonableness of the tertiary or quaternary structures. The register of the protein side chains to the map may be difficult to determine due to lack of visible landmarks such as large side chains. Additionally, starting models based on x-ray crystal structures of isolated domains or fragments may have errors, such as packing artefacts, that may not be resolved to the global energy minimum during refinement, instead getting trapped in a local energy minimum. Thus, a search method that quickly tests a large number of possible structures is needed. In our group, we have been using protein-protein docking to search billions of possible binding poses for a low-energy structure that fits the density map using ClusPro2.0 (Kozakov et al., 2017). These initial poses are ranked by an energy function containing terms such as attractive and repulsive van der Waals’ energy, electrostatic energy, and the desolvation energy. The representative structures from the largest clusters using the top 1,000 poses are then side-chain minimized to optimize the final energies in the output rankings. The top scoring poses, which can be calculated with a number of different weighting schemes, are then analyzed by the experimentalist to incorporate any available outside data (fit to an electron density map, mutational analysis, and known close residue-residue contacts) and the highest scoring pose(s) that satisfy the data are selected. As a check of the quality of the docked models, molecular dynamics simulations are performed to check the stability of model as well as fine tune structural interfaces by exploring conformations not tested during the rigid-body docking calculations.

**FIGURE 2:**
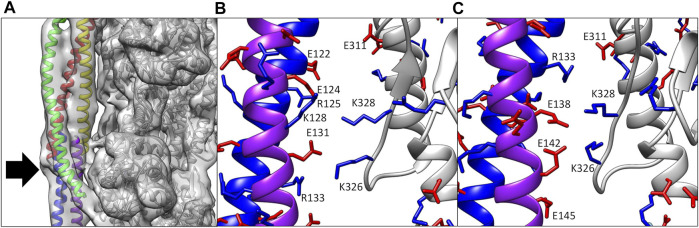
**(A)** Electron density map of the cardiac thin filament solved in calcium-free conditions. Note the tubular density for both tropomyosin (red, yellow, blue, and purple ribbon) and TnT1 (green ribbon), which could be fit by a number of potential structures. The only landmark here for the modeler is the N- and C-termini of tropomyosin (arrow). **(B)** Ca^2+^-bound model (Yamada et al., 2020) showing poor alignment of positive (blue sticks) and negative residues (red sticks) between actin (gray ribbon) and tropomyosin (blue and purple ribbons). **(C)** Ca^2+^-bound model (Pavadai et al., 2020) in a similar orientation as **(B)**. Note the improved alignment of charged residues after rebuilding.

Of particular interest here, the coiled-coil structure of tropomyosin appears in the maps as two intertwined tubes and the TnT domain (TnT1) that extends over the polymeric overlap domain of tropomyosin is also a simple tube (Figure 2A). By translating and/or rotating the helices in the tropomyosin or TnT1 models, a large number of models that satisfy the electron density map can be generated, and selection of the final model requires careful consideration. Using ideal models, secondary structure prediction, and protein-protein docking, we were able to refine the models of the TnT1/tropomyosin overlap domain (Pavadai et al., 2019) and the coiled coil of tropomyosin in thin filaments solved in calcium-free and calcium-bound conditions (Pavadai et al., 2020; Lehman et al., 2021).

### Tropomyosin Contacts With Actin in Cryoelectron Microscopy Structures

The solution of the cryoEM structure of the cardiac thin filament in calcium-free and calcium-bound states by Yamada et al. revealed many key relationships between tropomyosin, actin, and troponin. Analysis of the structure by our group, however, revealed unfavorable interactions between tropomyosin and actin in the Yamada modeling. The authors had started with a homology model of tropomyosin based on a low resolution (7 Å) crystal structure (Whitby and Phillips, 2000) whose coiled-coil pitch was longer than that present in the electron density maps [147 Å vs. 138 Å, as measured by the program Twister (Strelkov and Burkhard, 2002)]. In the final structure, both the end-to-end length of tropomyosin and the alpha helical rise were smaller than the expected values (see Table 4 in Pavadai et al., 2020). This may have been a result of compression of the helices during refinement as the coiled-coil pitch was reduced to match the maps. The result is that the tropomyosin present in these structures makes poor interactions with actin and cannot be extended into a polymeric cable.

We took two approaches to fitting tropomyosin into the electron density maps. First, we generated ideal, computer generated coiled-coil structures (Lorenz et al., 1995) with different rotations around their super-helical axis. Side chains in these models were derived from high resolution crystal structures of tropomyosin fragments (Li et al., 2010; Li et al., 2011; Rynkiewicz et al., 2015). After rigid body fitting of these models into the electron density maps in Chimera (Pettersen et al., 2004), the model that most closely matched the observed position of the C-terminus in the maps was selected for further fitting with molecular dynamics flexible fitting (MDFF) (Trabuco et al., 2008). The fitted coiled coil then had the previously published overlap domain/TnT1 model (Pavadai et al., 2019) grafted on to create the model used for molecular dynamics.

The above method does not account for any localized twisting, which can optimize tropomyosin/actin interactions (Lehman et al., 2018). Therefore, we used protein-protein docking to sample tropomyosin-actin interactions over a broader range of conformational space. We used a “divide and conquer” strategy whereby tropomyosin was divided into four pieces of about 60 amino acids in the middle of the molecule and an overlap domain of about 100 amino acids, each having about 20 amino acids overlapping with their neighboring segments. The list of poses docked to a model comprising two actin subunits was analyzed for fit to the electron density map, proper polarity, and overlap with neighboring poses. Through fusion of the overlapping regions in the various docked poses, the entire tropomyosin cable was built out and further fit into the cryo-EM density using MDFF, yielding a structure that has locally optimized interactions with actin. The models from the two methods are very similar, with both showing improved electrostatic interactions with actin (compare Figures 2B,C; see also Table 1 in Pavadai et al., 2020).

We also used this approach to model tropomyosin in the calcium-free (B-state) structure (Pavadai et al., 2020; Lehman et al., 2021), which shows similar compression in the tropomyosin helices as observed in the calcium-bound (C-state) structure. Again, we built models from ideal coiled coils and docking of smaller fragments of tropomyosin. Here, the docking models were key to guiding the building process, since the twist of tropomyosin is not equal across the entire molecule in the calcium-free maps, thus an ideal coiled coil structure does not fit in the map well without real space refinements. Analysis of the resulting models showed improvements to the interactions with actin, showing key salt bridges between actin residues Lys326, Lys328, and Asp311 and oppositely charged amino acids along the entire tropomyosin chain.

Lastly, the docking method was used to model tropomyosin in the M-state by fitting to the nucleotide-free rigor actomyosin-tropomyosin complex cryoEM map (Doran et al., 2020). This map also shows tropomyosin as two, intertwined tubes with no landmarks to guide positioning. Thus, assignment of the tropomyosin residues required the above approaches. The resulting model shows that tropomyosin movement from C-state to M-state is mostly a rotation around the actin helical axis, with little longitudinal movement or rotation around its own axis. The model also showed interactions between tropomyosin and loop four of myosin, notably between Arg369 and oppositely charged residues on tropomyosin.

### Tropomyosin Contacts With Troponin T and Troponin I in Cryoelectron Microscopy Structures

In addition to actin, tropomyosin also makes key interactions with TnT and TnI. TnT contains a TnT1 domain (residues 89–151 in cardiac TnT isoform 6) that is bound to the tropomyosin overlap domain in the cryoEM structure (Yamada et al., 2020), where it anchors the troponin complex to the thin filament, stabilizes the tropomyosin overlap domain, and maintains the 1:1 stoichiometry of troponin with tropomyosin in the thin filament (Flicker et al., 1982). Previously, a complex of peptides of TnT1 and the tropomyosin overlap domain had been solved in an x-ray crystal structure (Murakami et al., 2008). However, this crystal structure has poor density for the TnT and N-terminal tropomyosin peptides as well as a stoichiometry inconsistent with the thin filament. This crystal structure was used to guide the placement of the TnT1 domain by several groups (Manning et al., 2011; Gangadharan et al., 2017; Williams et al., 2018; Yamada et al., 2020) resulting in models that show poor contacts between TnT1 and tropomyosin. Thus, a method such as protein-protein docking, with a much larger exploration of conformational space and therefore a lower potential bias, seemed ideal for this system.

We started by docking alpha helical segments of TnT1 [residues 70–170 of cardiac TnT are ∼90% alpha helix as measured by circular dichroism (Palm et al., 2001)] to a model of the tropomyosin overlap domain based on the NMR solution structure and extended to a full filament (Greenfield et al., 2006; Orzechowski et al., 2014). The docking poses were analyzed to select poses that span the overlap in the expected antiparallel orientation to tropomyosin (Flicker et al., 1982; Cabral-Lilly et al., 1997; Jin and Chong, 2010). We used small, helical fragments with overlapping residues to extend TnT1 across the curved overlap domain of tropomyosin using multiple docking runs. The docking studies were terminated when high-scoring poses that could be reasonably added to the growing chain were predicted to make major clashes with actin.

In this way, cardiac TnT1 residues 89–151 and skeletal muscle TnT1 residues 79–141 were docked onto the tropomyosin overlap domain. The structures were similar with the exception of a pocket in the protein-protein interface of the cardiac TnT1/tropomyosin model not found in the skeletal muscle model. The models were stable in molecular dynamics simulations, with small fluctuations of the TnT1 termini. Consistent with the approximately 20 nM dissociation constant for TnT1/tropomyosin (Tobacman et al., 2002; Jin and Chong, 2010; Gangadharan et al., 2017), the cardiac TnT1 model showed a large, buried solvent-accessible surface area between the two proteins (an average of 2,295 Å^2^ during molecular dynamics) and many strong electrostatic interactions—representing an improvement over the previous models (Pavadai et al., 2020). After publication of the cryoEM maps from Yamada et al. (2020), we found that our TnT1/tropomyosin model had an excellent fit to the electron density map, thus cross-validating the two models.

The final part of the tropomyosin model to analyze was its interaction with the C-terminus of TnI. The C-terminus of TnI consists of several domains—the inhibitory peptide (residues 137–148 in cardiac TnI) which binds to actin, then the switch peptide (residues 149–163) which binds either actin or TnC in a calcium-dependent manner, and finally a H_4_-helix (residues 164–184) and C-terminal tail domain (residues 185–210) that bind actin in the calcium-free structure locking it in the sterically blocking position, but are unresolved and presumably disordered in the calcium-bound maps (Yamada et al., 2020). The latter three domains make potential calcium-dependent interactions with tropomyosin in the Yamada et al. structure; however, as discussed above, they were refined against a compressed tropomyosin. For example, analysis of the deposited coordinates show several charged tropomyosin residues (Arg133, Lys136, Glu138, Lys140 and Glu145) interacting with hydrophobic patch on TnI formed by residues 155–173, thus suggesting a modeling error.

After docking and refitting of the tropomyosin model, the TnI C-terminus was also docked to the model to check the proper placement of this domain. The refined structure shows hydrophobic residues on tropomyosin (Ala155, Ile154, Ala151, Leu148, Ile146, and Ile143) now in contact with TnI residues 155–173 (see Figure 3 in Lehman et al., 2021). Energy analysis of the tropomyosin/TnI contacts showed that most of the binding energy comes from association of the helix H_4_ and C-terminal domains with tropomyosin. On the other hand, most of the actin/TnI binding energy is contained in the inhibitory peptide.

## Discussion

As stated in the Introduction, accurate models of the thin filament are essential for understanding regulation of muscle contraction and early events in the development of inherited cardiomyopathies. Here, our group has used protein-protein docking methods along with molecular dynamics to refine cryoEM-derived models (Yamada et al., 2020). The regulatory function of the thin filament requires dynamic motions of tropomyosin/troponin and it is essential that the models accurately represent the key interactions of these proteins in their various structural states. The cooperative nature of the regulatory transitions suggests that long-range effects could be common for cardiomyopathy-causing mutations, therefore a globally accurate model of the thin filament is crucial for formation of mechanistic hypotheses. For example, in the hypertrophic cardiomyopathy tropomyosin mutant E192K (Sewanan et al., 2021), residue 192 was found to pass over a charged patch on actin that would be unfavorable for the mutant, potentially impairing tropomyosin’s ability to return to a sterically-blocking state. In the case of the dilated cardiomyopathy tropomyosin mutant M8R (Racca et al., 2020), the mutational effects in molecular dynamics simulations are local on the structure of the overlap domain, but there are also long-range shifts in the middle residues of tropomyosin to azimuthal positions predicted to enhance steric-blocking in low calcium conditions leading to hypocontractility. In this case, since the mutation is in the head-to-tail overlap domain, having proper helical structure in the tropomyosin model and coiled-coil helical symmetry matching that of actin was critical to accurately propagate mutational effects in the simulation both within and across tropomyosin dimers in the thin filament cable. Most recently, we have used the docking techniques discussed here to model the cardiac-specific N-terminus on TnI (Pavadai et al., 2022) which is the target of protein kinase A phosphorylation at serine residues 23 and 24. The model proposes that positively charged residues on tropomyosin, notably Arg 160, can interact with the phosphoserines, potentially biasing tropomyosin to a sterically blocking state and contributing to the observed increased relaxation rate found during adrenergic stimulation in the heart. Interestingly, E40K, E54K, and D230N mutations in cardiac tropomyosin have been shown to abolish cardiac TnI phosphorylation-dependent effects on calcium sensitivity *in vitro* assays of tissue-purified proteins (Memo et al., 2013), and the new model will help to understand the molecular basis of these observations. In the above examples, the derived mechanistic hypotheses critically rely on accurate representations of the thin filament proteins and their interactions along the entire length of the filament.

There are still some unresolved portions of the thin filament which may contribute to thin filament energetics, and these need to be accurately modeled to fully unlock the predictive power of the thin filament models. These include the linker region of TnT, which connects the TnT1 domain to the troponin core and the hypervariable N-terminus of TnT, an intriguing part of the thin filament, which may act as a calcium reservoir in insect muscle (Cao et al., 2020). These regions show weak or no density in the maps so they are presumably disordered, making modeling challenging. They could be represented by a small ensemble of structures generated using the computational techniques discussed here using biochemical data to guide the selection of the docking poses included in the final structure. The structures presented here could also be used as templates to make homology models of skeletal muscle thin filaments to propose mechanistic hypotheses explaining mutations that lead to skeletal myopathies. However, one must always inspect the intra- and intermolecular interactions formed by the proteins in the filament to ensure that these interactions are chemically reasonable and the resulting models are of the highest quality achievable.
